# EFFECTS OF A USER-CENTRED TECH-SUPPORTED TRANSITIONAL CARE MODEL ON REDUCING DEPENDENCY AMONG DISCHARGED PATIENTS

**DOI:** 10.1093/geroni/igae098.2448

**Published:** 2024-12-31

**Authors:** Nan Xia, Clio Yuen Man Cheng, Vivian Weiqun Lou

**Affiliations:** The University of Hong Kong, Hong Kong, China (People’s Republic); The University of Hong Kong, Hong Kong, China (People’s Republic); The University of Hong Kong, Hong Kong, China (People’s Republic)

## Abstract

Many patients, especially those aged above 60, continue to have care needs after discharge. However, the existing professional-led services do not meet the growing service needs in Hong Kong, especially during the Covid-19 pandemic. To fulfil the needs of discharged patients, a standardised transitional care service delivery model was established through a user-centred approach. A comprehensive one-stop online platform was developed to empower patients and their carers by enabling a better understanding of their conditions. The tech-supported solution also enabled service providers to deliver high-quality services through data-informed service plans without contact. The current study aims to evaluate the effects of the novel service delivery model on reducing dependency among discharged patients dwelling in the community. The interRAI-Home Care v9.3 was applied to collect information on patients’ dependency by evaluating their care needs before and after receiving the services. 431 subjects were recruited from three local NGOs between July 2021 and June 2023, and 286 (66.4%) participants completed both pre- and post-evaluations. The overall mean number of Clinical Assessment Protocols (CAPs) decreased significantly, from 7.4 to 5.5 (p < 0.001). Patients’ care needs were reduced significantly (p < 0.001) in each of the three institutions after receiving services, with no significant differences observed across institutions. The current study provides evidence of the effects of a user-centered tech-supported transitional care model for discharged patients and their carers, which ensures that such a model could effectively provide remote services to patients and their carers, and can facilitate alleviating manpower shortages while facing unexpected obstacles.

